# Supplementary material for: The effect of cyberbullying on nonsuicidal self-injury in adolescents: The chain mediating role of shame and dissociation
Source: PLoS One. 2026 Mar 27;21(3):e0345717. doi: 10.1371/journal.pone.0345717 (PMC13028519; doi:10.1371/journal.pone.0345717)
Supplement: Attachment — Submitted filename: Response to Reviewers.docx [file pone.0345717.s003.docx]

**Responses to Reviewers’ Comments**

**Manuscript ID:** [PONE-D-25-19748]
**Title:** [The effect of cyberbullying on nonsuicidal self-injury in adolescents: the chain mediating role of shame and dissociation]

We thank the reviewer for their thoughtful comments and constructive suggestions. We have addressed all points as detailed below. The changes in the manuscript are highlighted in yellow.

**Introduction**

****Comment 1：**** [ I wondered if there were any theoretical frameworks that might help support the association with shame and cyberbullying that could be integrated in the background. This might be helpful to also build into the discussion to inform different intervention strategies.]

****Response:**** We thank the reviewer for this insightful comment regarding the integration of theoretical frameworks. In response, we have incorporated a theoretical perspective to better substantiate the association between shame and cyberbullying. After reviewing the literature, we identified objectification theory as a relevant framework. This theory posits that individuals subjected to appearance-related cyberbullying may internalize external criticism and begin to perceive themselves from an observer’s perspective—a process known as self-objectification. This, in turn, can evoke feelings of shame rooted in the perceived failure to conform to socially prescribed appearance standards. We have integrated this theoretical explanation into the “The potential mediating role of shame” section of the manuscript (Page 4, Lines 74–80). We believe this addition strengthens the conceptual foundation of our study and offers valuable implications for shaping targeted intervention strategies.

****Comment 2:**** [ What drove the rationale to focus particularly on gender? It would be helpful to include this in the introduction.]

****Response:**We thank the reviewer for this constructive suggestion. In response, we have clarified the rationale for focusing on gender differences in the revised introduction. As summarized below, existing literature indicates that females are more likely to experience cyberbullying—particularly appearance-related attacks, which are strongly linked to body shame—and also report higher rates of NSSI. In contrast, males are more frequently identified as perpetrators of cyberbullying, while gender differences in dissociation remain inconclusive. Based on this evidence, we hypothesize that gender may meaningfully influence the pathway from cyberbullying to NSSI. These points have been incorporated into the manuscript on Page5-6, Lines 104–110.**

**Method/results**

****Comment 3:**** [ What demographic variables were measured? It would be helpful to outline them.This relates to my point below regarding at-risk subgroups - I wondered was any data collected on other psychological, behavioural, or sociodemographic characteristics that might enhance risk of or enhance the negative impact of cyberbullying? Especially when measuring NSSI. If not it would be helpful to discuss this as a limitation and area of further research.]

****Response:**We sincerely thank the reviewer for these insightful questions regarding demographic variables and potential risk factors. In response, we have now added a detailed description of the collected demographic information and conducted supplementary analyses to examine subgroup differences.**

**The demographic variables systematically collected in this study included: gender, grade level (10th, 11th, 12th grade), left-behind status (yes/no), and parental marital status (married/divorced/widowed). Subsequent analyses revealed significant associations between these factors and our key study variables:**

**• Grade Level: A one-way ANOVA showed statistically significant differences across grades for both cyberbullying and NSSI scores (p < 0.05). Post-hoc tests indicated that 10th graders reported the highest scores, which decreased significantly by 12th grade.**

**• Left-behind Status: Independent t-tests confirmed that left-behind students scored significantly higher on both cyberbullying (t = 72.04, p < 0.05) and NSSI (t = 3.77, p < 0.05) compared to their peers.**

**• Parental Marital Status: ANOVA results indicated significant group differences. Students with widowed parents reported the highest cyberbullying scores, while those with divorced parents showed elevated NSSI scores compared to students from intact families.**

**These comprehensive analyses have been incorporated into the manuscript on Page 7, Lines 140-142 and Page 10-11, Lines 214-225. We acknowledge the reviewer's valuable point about additional psychological or behavioral factors. While our study focused on core demographic variables, we agree that including other characteristics (e.g., socioeconomic status, pre-existing mental health conditions) would have provided deeper insights into at-risk subgroups. We have now explicitly discussed this as a study limitation and an important direction for future research in the revised discussion section.**

****Comment 4:**** [ Did any of the scales contain subscales that relate to the different forms of cyberbullying and different manifestations of shame? I wondered about the prevalence of different types of cyberbullying and shame and if it was possible to report prevalence across gender. The nature of cyberbullying and how shame manifests seems worth incorporating in the discussion as a point of further exploration especially considering intervention strategies.]

****Response:**** We sincerely thank the reviewer for this insightful suggestion regarding the analysis of subdimensions within the cyberbullying and shame scales. We fully agree that exploring different forms of cyberbullying and manifestations of shame—particularly in relation to gender differences—would add valuable depth to the study and strengthen the discussion on intervention strategies.

However, in preparing to conduct these analyses, we encountered a methodological limitation. Neither the original publications nor the available validation studies for the Revised Cyber Bullying Inventory (RCBI) and the Shame Scale for Secondary School Students provide detailed descriptions or clear operational definitions of their subdimensions. Despite a thorough review of the literature, we were unable to locate authoritative sources that would allow us to confidently classify items into specific subtypes (e.g., different forms of cyberbullying or categories of shame). Consequently, it was not feasible to analyze the prevalence of these subtypes or examine potential gender differences in their expression.

We acknowledge this as a limitation in the current study and have incorporated a discussion of this point into the limitations section of the manuscript (Pages 23, Lines 443–449). In future research, we plan to employ measurement tools with well-validated and clearly defined subscales to enable more nuanced investigations into the manifestations of cyberbullying and shame.

****Results****

****Comment 5:**** [Did age/year of study factor into the analysis? It is mentioned in the discussion, however I wondered if this was included in the analysis at any stage?]

****Response:**** We sincerely thank the reviewer for this valuable comment. In direct response, we have now conducted comprehensive analyses to examine the role of grade level, as well as other key demographic factors (left-behind status and parental marital status), in relation to our core variables. The detailed results have been added to the manuscript on **Page 10-11, Lines 214-225**.The specific findings are summarized as follows:

Grade-Level Differences: A one-way ANOVA revealed statistically significant differences across grades 10, 11, and 12 for both cyberbullying and non-suicidal self-injury (NSSI) scores (all *p* < .05). Post-hoc analyses confirmed that scores were highest in Grade 10 and lowest in Grade 12, indicating a clear decreasing trend as students progress through high school.

Cyberbullying: Grade 10 (M = 16.59, SD = 0.05) > Grade 12 (M = 15.61, SD = 0.05); F = 199.01.NSSI: Grade 10 (M = 2.23, SD = 0.10) > Grade 12 (M = 1.08, SD = 0.08); F = 166.63.

Left-Behind Status: Independent t-tests showed that students who were left-behind scored significantly higher on both cyberbullying (M = 16.50, SD = 4.00 vs. M = 16.20, SD = 3.70; t = 72.04, *p* < .05) and NSSI (M = 2.07, SD = 6.39 vs. M = 1.67, SD = 5.84; t = 3.77, *p* < .05) compared to their non-left-behind peers.

Parental Marital Status: Analysis of variance indicated significant effects of parental marital status. Students with widowed parents reported the highest cyberbullying scores, while students with divorced parents reported higher NSSI scores than those from families with intact marriages.

We acknowledge the reviewer's insightful point regarding the importance of controlling for these demographic factors. While these new analyses confirm that grade level and other background variables are significantly associated with our outcomes, they were not included as covariates in the primary correlation and mediation analyses presented in the initial submission. We would be pleased to conduct these additional supplementary analyses to control for these factors if the reviewer considers it essential for the robustness of our conclusions.

****Discussion****

****Comment 6:**** [ How does the prevalence in the current sample compare to other countries and cultures? It would be interesting to explore this.]

****Response:**** We thank the reviewer for this constructive suggestion. In response, we have added a comparative analysis of the prevalence rates of cyberbullying and non-suicidal self-injury (NSSI) across different cultural contexts and explored potential reasons for the observed variations. The key points of this discussion are as follows:

The detection rate of cyberbullying in our sample is consistent with rates reported among U.S. high school students prior to 2015. However, it is important to note that post-2015 estimates from the U.S. show considerable variability, ranging from 3% to 72% , highlighting the challenge of direct comparison due to methodological differences.

The prevalence observed in our study was higher than the lifetime cyberbullying rate of 7.02% reported among Australian children and adolescents. These discrepancies may be attributed to cross-cultural variations in the definition of cyberbullying and the lack of standardized measurement tools.

Region-specific factors may also play a role. In China, the highly competitive exam-oriented education system imposes significant academic pressure on high school students, leaving limited avenues for recreation. Consequently, many turn to the internet as a primary means of stress relief. Increased online exposure is a well-established risk factor correlated with higher rates of cyberbullying victimization.

Similarly, our observed NSSI prevalence of 25.4% was higher than the pooled community prevalence of 17.6% reported in a meta-analysis of 17 countries, including Canada and the U.S. Within the Chinese cultural context, where direct expression of psychological distress is often discouraged, adolescents may be more inclined to internalize emotional pain and express it through somatic or behavioral channels, such as self-injury, rather than through verbal communication.

These modifications have been incorporated into the manuscript on pages 17 (Lines 310–322) and pages 18 (Lines 335–340). We believe this discussion significantly strengthens the cross-cultural relevance of our findings.

****Comment 7:**** [I wondered if there is further evidence to suggest who is at most risk of cyberbullying or who may be prone to the enhanced risk of the negative impact of bullying in certain subgroups of high school students (for example those who are neurodiverse, ethnically diverse, live with a disability or long-term mental or physical health condition?) Lines 324 - 327 elude to this, as well as the discussion of temperament and coping tendencies. ]

****Response:****We thank the reviewer for raising this critical and insightful question regarding the identification of subgroups at heightened risk for cyberbullying involvement and its negative impacts. This is indeed a crucial area for developing targeted interventions.We agree with the reviewer that a more detailed discussion of vulnerable subgroups is essential. In our original manuscript, we alluded to this in the context of temperament and coping (Lines 324-327), but we acknowledge that a more explicit and evidence-based exploration was warranted.In response to this comment, we have now:Revised the Introduction/Literature Review section to explicitly mention the established risk factors and vulnerable subgroups at the outset.Substantially expanded the Discussion section to dedicate a new paragraph to discussing the evidence for these subgroups, incorporating key references as suggested below.Added a consideration of "intersectionality" to note how overlapping identities.We believe these changes significantly strengthen the paper by providing a more nuanced understanding of cyberbullying vulnerability, as requested by the reviewer. The new text can be found into lines 50-53 on page 3 and lines 404-425 on page 21-22 of the original manuscript.
