# Supplementary material for: The effect of cyberbullying on nonsuicidal self-injury in adolescents: The chain mediating role of shame and dissociation
Source: PLoS One. 2026 Mar 27;21(3):e0345717. doi: 10.1371/journal.pone.0345717 (PMC13028519; doi:10.1371/journal.pone.0345717)
Supplement: Attachment — Submitted filename: Responses to Reviewers.docx [file pone.0345717.s004.docx]

Responses to Reviewers' Comments

Manuscript ID: PONED2519748

Title: The Effect of Cyberbullying on Nonsuicidal SelfInjury in Adolescents: The Chain Mediating Role of Shame and Dissociation

We sincerely thank the reviewers for their thoughtful comments and constructive suggestions, which have been invaluable in strengthening our manuscript. We have carefully addressed each point as detailed below. All revisions are highlighted in green within the revised manuscript.

**Major Comments**

1. Causal language must be revised. The manuscript repeatedly implies causal pathways (e.g., “cyberbullying leads to…”, “shame mediates…”). Given the crosssectional design, authors must use noncausal phrasing such as “is associated with” or “statistically mediates.”

Response: We fully agree with the reviewer. The crosssectional nature of our data does not permit causal inferences. Accordingly, we have systematically revised the manuscript to replace all causal language with associative or correlational terminology.

Changes made:

Abstract: "shame and dissociation have a mediating role" was revised to "shame and dissociation played a mediating role" (P2, L28); "can increase the nonsuicidal selfinjury risk" was revised to "synergistic effects of shame and dissociation may be associated with increased risk of adolescent nonsuicidal selfinjury" (P2, L35–36).

Introduction: "makes shame a crucial emotion" (P4, L83) was revised to "positions shame as a crucial emotion"; "Increasing shame results in……" was revised to "Increased shame is associated with" (P5, L100–101).

Results: In descriptions of regression models (e.g., Table 3 caption and text), we consistently used "was associated with" rather than "impacted" or "influenced." The heading "Chain mediated effects test" was revised to "Analysis of the mediating roles."

Discussion: All causal expressions have been modified to noncausal phrasing throughout. Terms such as "pathway," "link," and "association" are emphasized, and phrasing like "may contribute to" or "could be linked to" is used to describe potential mechanisms.

Conclusions: "providing new ideas for intervening" was tempered to "offers insights potentially relevant for intervention development" (P24, L488).

2. Justification for covariates is insufficient. Since grade, leftbehind status, and parental marital status significantly differ across main variables, authors should either: include them as covariates in mediation models, or provide a clear theoretical and methodological rationale for excluding them.

Response: We appreciate this important observation. Upon reconsideration, we agree that incorporating these significant demographic variables as covariates will enhance the robustness of our findings. We have therefore added grade level, leftbehind status, and parental marital status as covariates in all mediation analyses and have updated the corresponding results accordingly. This revision accounts for potential confounders, thereby providing more precise estimates of the relationships between cyberbullying, the mediators, and NSSI.

Changes made:

Results Section: All references to mediation analyses now explicitly state that the models controlled for the expanded set of covariates: "...after controlling for gender, traditional bullying victimization, grade, leftbehind status, and parental marital status." The results (beta coefficients, confidence intervals, effect ratios) have been updated accordingly in the text and tables. The fundamental pattern of significant mediation effects remained stable.

Footnote to Tables 3 & 5: A standard note was added: "All models controlled for gender, traditional bullying victimization, grade, leftbehind status, and parental marital status."

3. Interpretation of effect sizes is needed. Some statistically significant paths $\text{e}\text{.}\text{g}\text{.,}\text{β}\text{=.03}$ are small. Authors should explicitly discuss practical or clinical significance and the contribution of the large sample size to statistical significance.

Response: We appreciate this suggestion. We have added explicit commentary on the interpretation of effect sizes, distinguishing statistical from practical significance, and acknowledging the role of the large sample size. This revision provides a more nuanced interpretation of the findings, cautioning against overvaluing statistically significant but small effects while appropriately contextualizing the results within the largescale study design.

Changes made:

Results Section (P15, L293–298): A new paragraph was added: "While the standardized coefficients for some paths (e.g., shame → NSSI, β = .03) were small, they reached statistical significance due to the large sample size. The indirect effect via dissociation was substantially larger than that via shame. This indicates that, although all tested pathways are statistically reliable, dissociation may represent a more prominent psychological mechanism in the association between cyberbullying and NSSI in this sample. The clinical relevance of these small effects, particularly in the context of prevention, requires further investigation."

Discussion Section (P22, L422–425): We added: "It is important to note that the mediation effect for shame, while statistically significant, was small in magnitude. This suggests that although shame plays a role, its direct associative contribution to NSSI in the context of cyberbullying may be limited relative to other factors such as dissociation."

4. a) The lack of subscale definitions for shame and cyberbullying measures limits nuanced interpretation.

b) The NSSI severity composite (frequency × injury severity) requires stronger justification or citation validating this scoring method.

c) Clarify psychometric properties beyond Cronbach’s α (e.g., factor structure).

Response: We thank the reviewer for highlighting these important methodological details. We have expanded the discussion of measurement limitations and provided additional justification. These additions enhance methodological transparency, acknowledge the constraints of the chosen analytical approach, and offer clear directions for future research.

Changes made:

4a & 4c – Measures Section (P10, L200–203): For the Shame Scale and Revised Cyber Bullying Inventory, we added: "Although both the Shame Scale for Secondary School Students and the Adolescent SelfInjurious Behavior Questionnaire demonstrated good reliability, this study was unable to conduct separate analyses of their subscales due to the lack of detailed operational definitions and validated factor structures for these subcomponents in the existing literature."

4b – Measures Section (P9–10, L193–197): For the NSSI questionnaire, we added justification for the composite score: "The composite score (frequency × severity) was calculated to reflect the overall burden of NSSI behavior, a method used in prior adolescent NSSI research to capture both behavioral frequency and physical consequences. This approach provides a single index that accounts for both dimensions of selfinjurious behavior."

Limitations Section (P23, L461–466): We significantly expanded the discussion: "Third, measurement limitations warrant consideration. The use of total scores from shame and cyberbullying scales, without analysis of specific subdimensions (e.g., body shame vs. characterological shame; harassment vs. exclusion), limits our ability to identify more precise associative pathways. Future studies should employ measures with wellvalidated subscales to explore these nuanced relationships. Furthermore, while the composite NSSI score is informative, its specific weighting (frequency × severity) requires further validation."

5. Clarify ethical procedures. Provide a clear description of how consent/assent was obtained from minors, and how voluntariness and confidentiality were preserved in school settings.

Response: We have expanded the description of ethical procedures to address these crucial points. The revised text provides a clear, detailed account of the ethical safeguards implemented, ensuring compliance with standards for research involving minors.

Changes made:

Procedure Section (P7, L128–137): The paragraph was revised to: "This study was approved by the Ethics Committee of Mianyang Third People's Hospital (Approval Document No: 2022 Annual Review [10]). The study obtained passive parental consent and active student informed consent. Prior to data collection, a written study description was sent to parents/guardians, who could opt their children out of the study by returning the form. On the survey day, students received a detailed information sheet explaining the study's purpose, the voluntary nature of participation, anonymity, and the right to withdraw at any time without consequences. All participating students signed a written consent form. The survey was administered by trained researchers in a controlled classroom setting, with emphasis on the confidentiality of responses. Questionnaires were completed anonymously and collected separately from any identifying information. Data were stored on a passwordprotected secure server accessible only to the research team."

6. Discussion occasionally overinterprets cultural explanations. Statements linking cultural norms to NSSI prevalence should be supported by citations or phrased cautiously as hypotheses.

Response: We agree and have moderated the cultural interpretations accordingly. We have added supporting citations and reframed statements as speculative where direct evidence is lacking. This revision presents cultural explanations more cautiously and academically, framing them as plausible hypotheses within a broader empirical context rather than definitive conclusions.

Changes made:

Discussion Section (P19, L354–357): The sentence comparing NSSI prevalence was revised to: "Moreover, the prevalence in our sample exceeded the pooled rate of 17.6% reported in a 17country metaanalysis including Canada and the United States [56]. It has been suggested that in cultural contexts where overt expression of psychological distress is discouraged, adolescents may manifest internalized turmoil through behaviors such as NSSI [57, 58]. This dynamic may partially account for crosscultural differences, though direct comparisons remain complex."

We reviewed the entire Discussion to ensure similar statements were modified to use tentative language ("may," "could," "it is possible that") or were supported by citations.

Minor Comments

7. Improve English expression and correct grammatical issues throughout.

Response: The manuscript has undergone comprehensive proofreading and language editing by Editage (www.editage.com) to correct grammatical errors, optimize writing flow, and enhance clarity of expression.

8. Ensure consistent terminology (e.g., “traditional bullying score” vs. “traditional bullying victimization”).

Response: We have standardized terminology throughout the manuscript. When referring to concepts or variables, we now uniformly use "traditional bullying victimization." The term "traditional bullying score" is used specifically to denote the reported scale scores (as shown in Table 1). This consistency is maintained across the entire text.

9. Table labels could be clearer; consider adding effect sizes (Cohen’s d) for gender and NSSI comparisons; figure captions should summarize the key coefficients.

Response: We appreciate these helpful suggestions, which have improved the clarity of our tables and figures. Based on these comments, we have made the following revisions:

Table 1: The title has been revised to "Differences in Key Variables by Gender and NonSuicidal SelfInjury (NSSI) Status" to more accurately reflect the content. We have added a column for Cohen's d effect sizes for both gender comparisons and NSSI group comparisons, providing a more comprehensive understanding of the magnitude of observed differences beyond statistical significance alone.

Table 2: The title has been updated to "Descriptive Statistics and Bivariate Correlations of Primary Variables" to better reflect the presented data.

Figure 1 (Chain Mediation Model): The caption has been expanded to include key statistical information. The revised caption reads: "Chain mediation path model of the relationship between cyberbullying victimization, shame, dissociation, and nonsuicidal selfinjury (NSSI). Standardized path coefficients (β) are shown for each significant path. All analyses controlled for gender, traditional bullying victimization, grade level, leftbehind status, and parental marital status. Model fit indices: R = 0.45, R² = 0.21, MSE = 0.80, F(8, 14027) = 452.37, p < 0.001. p < 0.001."

Figure 2 (Parallel Mediation Model): Similarly, the caption has been revised to: "Parallel mediation path model of the relationship between cyberbullying victimization, shame, dissociation, and nonsuicidal selfinjury (NSSI). Standardized path coefficients (β) are shown for each significant path. All analyses controlled for gender, traditional bullying victimization, grade level, leftbehind status, and parental marital status. Model fit indices: R = 0.37, R² = 0.14, MSE = 0.86, F(6, 14029) = 370.88, p < 0.001. p < 0.001."

10. Improve clarity and flow in the Introduction; some paragraphs can be tightened for readability.

Response: The Introduction has been edited for conciseness and flow. Redundant phrases were removed, long sentences were split, and logical transitions between paragraphs were strengthened to improve readability and argument progression.

**Closing Statement**

The reviewers' and editors' constructive feedback has been invaluable in helping us refine this manuscript. We have carefully addressed each comment to the best of our ability and believe the revised version reflects these improvements. We fully recognize that further refinements may still be necessary, and we welcome any additional guidance to ensure the manuscript meets the rigorous standards of PLOS ONE. Thank you for your continued support and consideration.
